# Supplementary material for: The Therapeutic Treatment with the GAG-Binding Chemokine Fragment CXCL9(74–103) Attenuates Neutrophilic Inflammation and Lung Dysfunction during Klebsiella pneumoniae Infection in Mice
Source: Int J Mol Sci. 2022 Jun 2;23(11):6246. doi: 10.3390/ijms23116246 (PMC9181286; doi:10.3390/ijms23116246)
Supplement: Supplementary file 1 [file ijms-23-06246-s001.zip › ijms-1707341-supplementary.pdf]

Supplementary material:

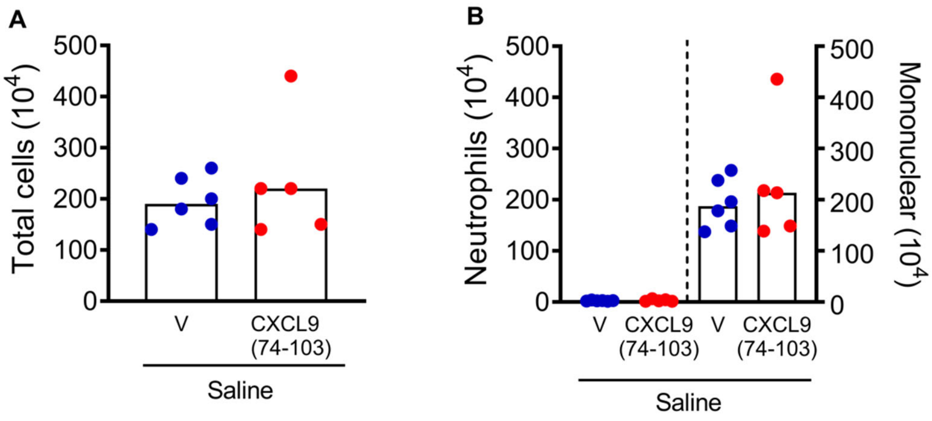

Figure S1: The treatment with CXCL9(74-103) has no effect on cell recruitment to airways in mice injected with saline. Mice received intratracheal injection of saline and after 6 hours mice were treated intravenously with 100 µL of CXCL9(74-103) 1mg/mL or vehicle (PBS). Mice were euthanized 24 hours after intratracheal challenge and (A) number of total leukocytes, (B) neutrophils and mononuclear cells was evaluated in BALF. Blue points= vehicle; red points= CXCL9 (74-103). Data are shown as median from one representative experiment; n= 6-5 mice per group.
